# Invasive fungal infection in liver transplant recipients in a prophylactic era: A multicenter retrospective cohort study in Korea

**DOI:** 10.1097/MD.0000000000016179

**Published:** 2019-06-28

**Authors:** Youn Jeong Kim, Sang Il Kim, Jong Young Choi, Seung Kew Yoon, Gun Hyung Na, Young Kyoung You, Dong Goo Kim, Myoung Soo Kim, Jae Geun Lee, Dong Jin Joo, Soon Il Kim, Yu Seun Kim, Sang-Oh Lee, Shin Hwang, Eungeol Sim

**Affiliations:** a Department of Internal Medicine, Incheon St. Mary's Hospital, The Catholic University of Korea; b Department of Internal Medicine, Seoul St. Mary's Hospital; c Department of Surgery, Seoul St. Mary's Hospital, The Catholic University of Korea; d Department of Surgery, Severance Hospital, Yonsei University College of Medicine; e Department of Internal Medicine, Asan Medical Center, University of Ulsan College of Medicine; f Department of Surgery, Asan Medical Center, University of Ulsan College of Medicine; g Merck Sharp & Dohme, Korea Ltd.

**Keywords:** fungal infection, liver transplant, prophylaxis, risk factor

## Abstract

The epidemiology of invasive fungal infections (IFIs) after liver transplantation (LT) is continuing to evolve in the current era of antifungal prophylactic therapy. This multicenter retrospective cohort study aimed to evaluate the epidemiology, risk factors, and outcomes of IFIs among LT recipients in the current era.

We analyzed a total of 482 LT recipients aged 18 years and older who were admitted to 3 tertiary hospitals in Korea between January 2009 and February 2012.

Twenty-four episodes of IFIs occurred in 23 patients (4.77%; 23/482). Of these episodes, 20 were proven cases and 4 were probable cases according to EORTC/MSG criteria. Among these cases, IFI developed within 30 days of transplantation in 47.8% of recipients, from 31 to 180 days in 34.8% of recipients, and from 181 to 365 days in 17.4% of recipients. The most common isolates were *Candida* species (n = 12, 52.2%; *Candida albicans*, 6 cases; *Candida tropicalis*, 1 case; *Candida glabrata*, 1 case; *Candida parapsilosis*, 1 case; and unspecified Candida species, 1 case) and *Aspergillus* species (n = 7, 30.4%). The mortality in patients with IFIs was significantly higher than that in patients without IFIs (47.83% [11/23] vs 7.18% [33/459], *P* < .001). The incidence of late-onset IFIs is increasing in the antifungal prophylactic era, and fluconazole-resistant non-*albicans Candida* species have not yet emerged in Korea.

## 1 Introduction

Infection after transplantation is a major factor affecting the outcome of transplant recipients. Although the incidence of invasive fungal infections (IFIs) is not higher than the incidence of bacterial infections among solid organ transplant recipients, IFIs are associated with high mortality of 65% to 90% for invasive aspergillosis and 30% to 50% for candidiasis.^[1,2]^

Advances in the diagnosis of IFIs, improvement in immunosuppressive agents, and refinement of surgical techniques have resulted in a decline in the incidence of post-transplant IFIs since the mid-1990s.^[3]^ The Infectious Disease Society of America guidelines in 2009 recommended antifungal prophylaxis for high-risk liver transplant (LT) recipients, and some studies have showed that antifungal prophylaxis among LT recipients has reduced the incidence of IFIs.^[4–6]^ In Korea, a consensus for antifungal prophylaxis among LT patients has not been achieved. Antifungal prophylaxis was rarely used due to insurance problem, drug toxicity, resistance, breakthrough infection, and cost-effectiveness. However, recently, practices of prophylactic therapy have been changing, and the studies on fungal infections in LT recipients are continuing to evolve.

Several preoperative, perioperative, and postoperative risk factors have been associated with IFIs and mortality among LT recipients.^[2,4,5]^ Identifying risk factors for IFIs can guide the timely use of prophylactic agents or early treatment of IFI among high-risk LT recipients, potentially improving the outcomes for LT patients. In Korea, there are limited center-specific studies in the epidemiology of IFIs and risk factors among LT recipients. The aim of this multicenter retrospective cohort study was to examine the incidence of IFI, risk factors, and clinical outcomes among LT patients in Korea 1 year after LT in the era of anti-fungal prophylaxis.

## 2 Material and methods

### 2.1 Study design and patients

The electronic medical records from 3 tertiary teaching hospitals in Korea were reviewed for all LT recipients over 18 years of age between January 2009 and February 2012. We excluded patients diagnosed with IFI at the time of transplantation. Diagnosis of IFI included individuals that met any of the proven, probable, or possible cases of European Organization for Research and Treatment of Cancer/Mycoses Study Group (EORTC/MSG) consensus criteria for IFI diagnosis.^[6]^

### 2.2 Definition

IFI was defined according to EORTC/MSG consensus.^[6]^ Fungal colonization was defined as the presence of fungus in the absence of clinical symptoms or evidence of infection within 3 months of transplantation. Prior use of an antifungal agent was defined as administration of antifungal agents within 3 months of transplantation.

### 2.3 Prophylactic strategy

Prophylactic antibiotics for LT were different for individual cases. Decision for antifungal prophylaxis was physician dependent.

### 2.4 Data collection

We reviewed medical records for demographic characteristics as well as preoperative, surgical and postoperative factors. We evaluated the medical records information regarding fungal infections including etiology and sites of infection within one year after transplantation through clinical data, microbiologic data, histopathologic data, radiologic data, and serologic data. Preoperative factors included clinical data (donor type, age, body mass index (BMI), model for end-stage liver disease (MELD) score, child Pugh score, antibiotic prophylaxis, dialysis, and indication for transplant) and laboratory data within 7 days prior to the liver transplantation. Fungal colonization was defined as the growth of fungus in clinical samples before transplantation without clinical signs or symptoms of infection. Surgical factors included operation time (min), intraoperative packed RBC transfusion volume (Unit) and anhepatic time (min). Postoperative factors included intravenous hyperalimentation, mechanical ventilation, presence of CMV infection, acute rejection and presence of IFI.

### 2.5 Outcomes

The primary endpoint of this study was to identify the incidence of IFIs after LT. We used 1-year mortality after liver transplant as a secondary outcome.

### 2.6 Ethics statement

This study was approved by the institutional review board of each hospital. All the data collected during this study were kept confidential.

### 2.7 Statistical analysis

Student's *t* test or the Mann–Whitney *U* test was used for analysis of continuous variables, and the X^2^ test or Fisher exact test was used for categorical variables. We analyzed the risk factors associated with IFIs and mortality using univariate and multivariate methods. The final model was determined by a stepwise variable selection method. Survival curves were generated using the Kaplan–Meier method with log-rank test. Statistical analyses were performed using SAS Software version 9.2. A *P* value <.05 was considered statistically significant.

## 3 Results

### 3.1 Baseline characteristics

Of 487 patients screened in this study, 5 patients were excluded; 2 patients were younger than 18 years old at the time of transplantation and 3 patients had preoperative IFIs. The demographic characteristics are shown in Table 1. The median age was 53 years (range 23–69 years), and 76.8% of patients (n = 370) were male. Diabetes mellitus was the most common comorbidity (n = 119, 24.8%) followed by hypertension (n = 100, 20.8%). Liver cirrhosis (n = 409, 84.8%) was the most common indication for transplantation and 51% of patients had hepatocellular carcinoma. Retransplantation was performed in 1.9% of patients (n = 9). The median MELD score was 14 (range 6–40), and a Child Pugh score C was found in 36.8% (n = 175) of patients.

**Table 1:**
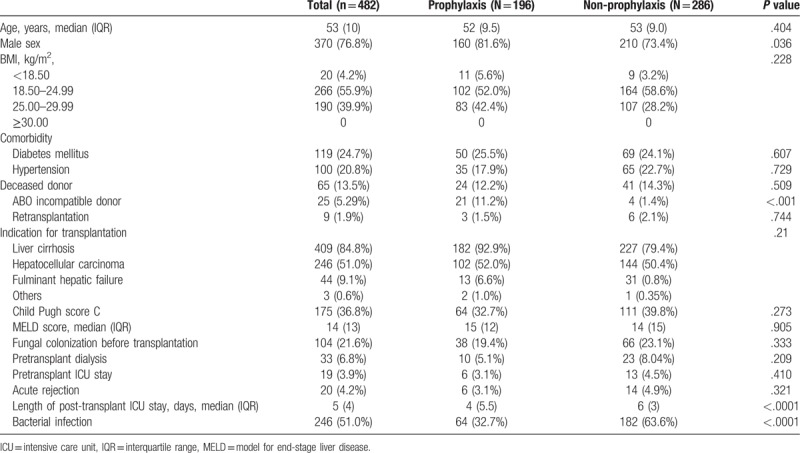
Demographic and clinical characteristics.

### 3.2 Incidence of fungal infection

Among 482 patients, IFIs occurred in 23 patients (4.8%; 23/482) with 24 episodes, of which 20 were proven cases and 4 were probable cases according to EORTC/MSG criteria. The most common etiology for IFI was *Candida* species in 12 patients (52.2%; *Candida albicans*, 6 cases; *Candida tropicalis*, 1 case; *Candida glabrata*, 1 case; *Candida parapsilosis*, 1 case; and unspecified *Candida* species, 1 case). *Aspergillus* species were found in 7 patients (30.4%) including 3 cases of *Aspergillus fumigatus*. Cryptococcus was isolated in 1 patient. The sites affected by IFI were the intra-abdominal space (n = 9, 39.1%), the lung (n = 8, 34.8%), catheter (n = 6, 26.1%), urinary tract (n = 2, 8.7%) and skin and soft tissues (n = 1, 4.4%).

Median time to first IFI after LT was 38 days (range 2–339 days). Development of IFI occurred within 30 days after transplantation in 47.8% (n = 11) of recipients, from 31 to 180 days in 34.8% (n = 8) of patients and from 181 to 365 days in 17.4% (n = 5) of patients. The causative organisms of IFIs according these time periods are summarized in Table 2.

**Table 2:**
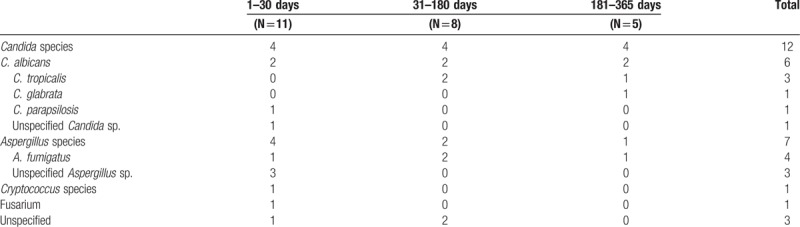
Etiological Organisms according to period after transplantation.

### 3.3 Risk factors for IFI

In univariate analysis, the risk factors associated IFI were prior use of antifungal therapy (OR 13.62, 95% CI 3.04–61.03, *P* = .0006), pretransplant dialysis (OR 5.28, 95% CI 1.48–12.37, *P* = .007), high MELD score (OR 5.93, 95% CI 1.48–23.71, *P* = .01), intraoperative RBC≥14 units (OR 2.23, 95% CI 0.95–5.22, *P* = .06), anhepatic time≥122 minutes (OR 3.80, 95% CI 1.12–12.89, *P* = .03), CMV infection (OR 4.12, 95% CI 1.71–9.92, *P* = .001), fungal colonization (OR 15.61, 95% CI 5.64–43.22, *P* < .0001), bacterial infection (OR 6.04, 95% CI 1.91–19.08, *P* = .002) and hyperalimentation (OR 3.80, 95% CI 3.00–17.69, *P* < .0001) (Table 3). Multivariate analysis showed that independent risk factors for IFI were history of antifungal agents (OR 28.47, 95% CI 3.33–243.7, *P* = .002), retransplantation (OR 15.69, 95% CI 2.08–118.52, *P* = .007), fungal colonization (OR 14.89, 95% CI 4.07–54.42, *P* = .007), and hyperalimentation (OR 6.2, 95% CI 2.1–17.70, *P* = .0009) (Table 3).

**Table 3:**
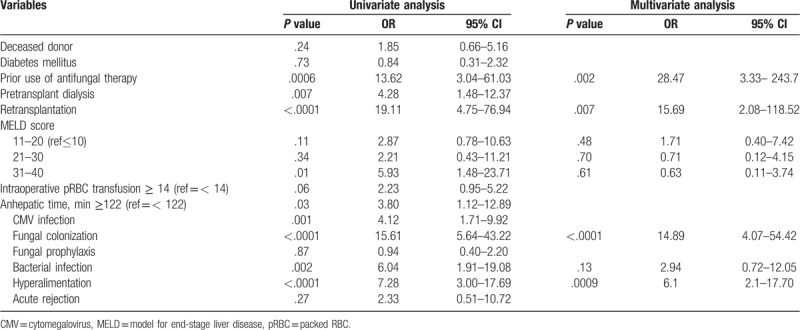
Risk factors for invasive fungal infections.

### 3.4 Secondary outcomes

**Figure 1:**
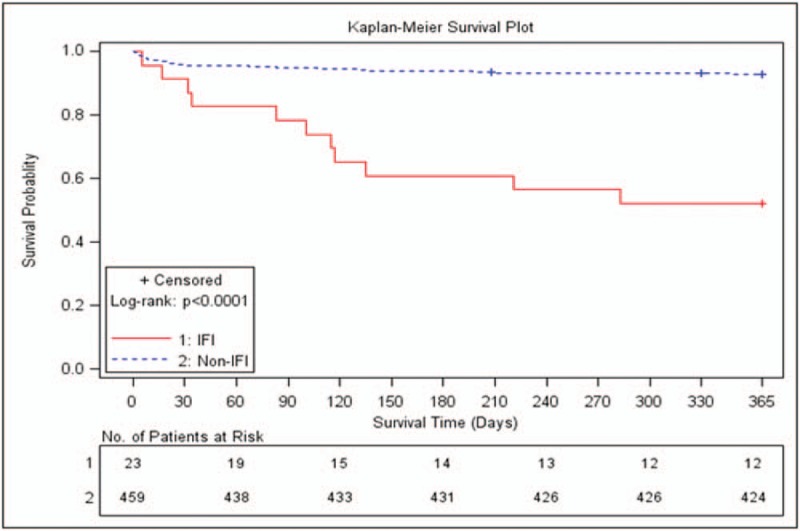
Kaplan-Meier survival curves for patients with and without IFI.

The 1-year overall mortality after transplantation was 9.13% (n = 44), and infection-related mortality was 4.9% (n = 24). Four patients experienced fungus-related deaths. The mortality in patients with IFIs was significantly higher than in those without IFIs (47.83% [11/23] vs 7.18% [33/459], *P* < .0001). The Kaplan-Meier survival curves for patients with IFI and those without IFI are shown in Figure 1.

### 3.5 Antifungal prophylaxis and outcomes

**Figure 2:**
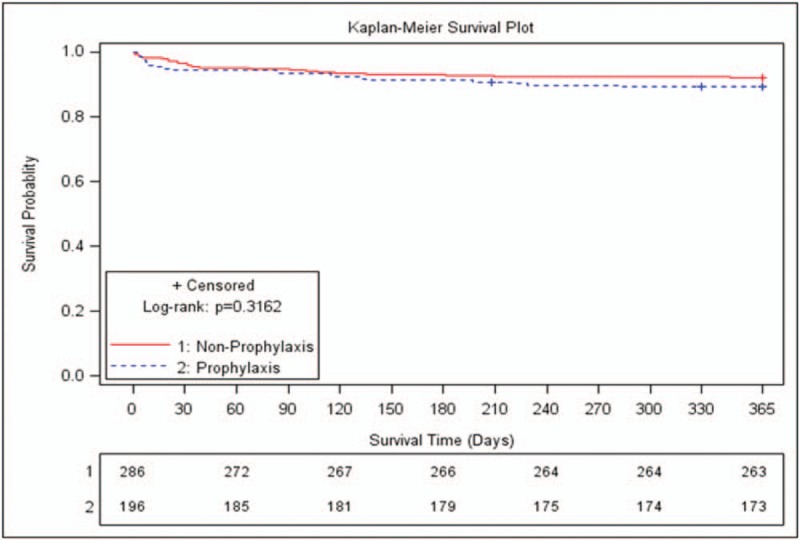
Kaplan-Meier survival curves stratified by use of antifungal prophylaxis.

Systemic antifungal prophylaxis was administrated in 40.6% (n = 196) of a total of 482 patients. IFI was found in 9 patients in antifungal prophylaxis groups and 14 patients in non-antifungal prophylaxis group (*P* = .87). There were more ABO-incompatible donors in prophylaxis group (11.2% [21/196] vs 1.4% [4/286], *P* < .001). Recipient age (52 years (IQR 9.5) vs 53 years (IQR 9.0), *P* = .404), MELD score (15 (IQR 12) vs 14 (IQR 15), *P* = .91), Child Pugh score C (32.7% [64/196] vs 39.8% [111/286], *P* = .273) and fungal colonization (19.4% [38/196] vs 23.1% [66/286], *P* = .333) were not different between the 2 groups (Table 1). Patients in prophylaxis group had shorter duration ICU stays post-transplantation (4 days (IQR 5.5) vs 6 days (IQR 3), *P* < .0001) and developed bacterial infection less frequently (32.7% [64/194] vs 63.6% [182/286], *P* < .0001) than those in non-prophylaxis group. However 1-year overall mortality was not different between the 2 groups (10.8% [21/194] vs 8.0% [23/286], *P* = .30) (Fig. 2). The median time to the first occurrence of IFI after LT was 79 days (IQR 66) in the prophylaxis group and 22.5 days (IQR 48) in the non-prophylaxis group (*P* = .643). The rate of first IFI occurrence within 90 days of transplantation was not statistically different between the 2 groups (66.7% [6/9] vs 78.6% [11/14], *P* = .643)

### 3.6 Risk factors for mortality

Univariate analysis revealed that the risk factors associated with mortality were deceased donor organ transplant (OR 5.23, 95% CI 2.87–9.55, *P* < .0001), preoperative dialysis (OR 6.60, 95% CI 3.39–12.82, *P* < .0001), MELD score, presence of IFI (OR 7.74, 95% CI 3.90–15.34, *P* < .0001), bacterial infection (OR 6.29, 95% CI 2.66–14.88, *P* < .0001), hyperalimentation (OR 2.41, 95% CI 1.32–4.40, *P* = .004), retransplantation (OR 4.50, 95% CI 1.39–14.55, *P* = .01), fungal colonization (OR 2.87, 95% CI 1.58–5.21, *P* = .0005), acute rejection (OR 3.14, 95% CI 1.24–7.96, *P* = .02), CMV infection (OR 2.20, 95% CI 1.13–4.27, *P* = 0.02) and use of mechanical ventilation (OR 3.67, 95% CI 1.14–1.86, *P* = .02) (Table 4). Multivariate analysis revealed that deceased donor (OR 13.00, 95% CI 5.1–33.1, *P* < .0001), presence of IFI (OR 6.72, 95% CI 2.62–17.22, *P* < .0001), bacterial infection (OR 2.83, 95% CI 1.10–7.26, *P* = .0001) and hyperalimentation (OR 3.45, 95% CI 1.51–7.85, *P* = .003) were independent risk factors for mortality after transplantation (Table 4).

**Table 4:**
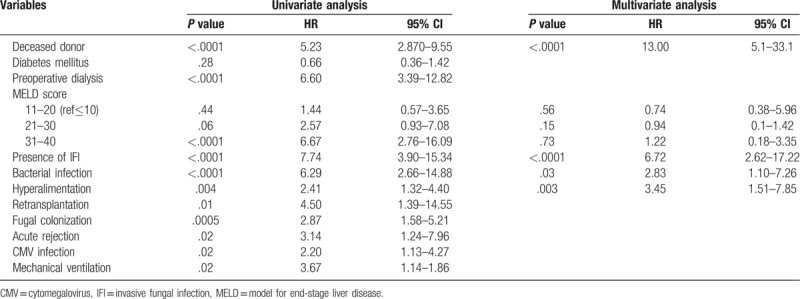
Risk factors for mortality.

## 4 Discussion

Fungal infection is associated with high morbidity and mortality among patients with LT.^[7,8]^ The present retrospective study from 3 tertiary hospitals in Korea found that IFIs developed in 4.8% of LT recipients. The mortality rate in patients with IFIs after LT approached 50%. Recently, the incidence of IFIs has decreased because of improvement in surgical techniques, advances in immunosuppressive agents, and better antifungal prophylaxis.^[7,9,10]^ The incidence of IFI is reported to be 7% to 42% in solid organ transplant recipients and in our previous single center study, we reported that the rate of fungal infection after LT was 5.4%.^[1,11–13]^ Our study revealed a trend towards a slightly lower incidence of IFI compared to our previous findings. No standardized protocol for antifungal prophylaxis in LT recipients was in place as shown in our previous study, and antifungal prophylaxis in transplant recipients was not common practice in most centers in Korea.^[11]^ However, recently, there has been an increasing trend toward treatment using antifungal prophylaxis in solid organ transplant recipients in Korea. In this study, about 40% of the patients underwent systemic antifungal prophylaxis despite the limitation of insurance policies regarding the use of antifungal prophylaxis in Korea. Our study showed that the patients who received antifungal prophylaxis had a higher percentage of ABO incompatible donors and post-transplant bacterial infection. These findings suggest that antifungal prophylactic strategies have shifted to target high-risk LT patients in Korea. In addition, we observed that about 18% of IFIs developed in recipients after 6 months of transplantation, while the rest developed within 6 months of transplantation. In our previous study, only one case of an IFI had developed after 6 months of transplantation in LT patients without antifungal prophylaxis.^[11]^ In general, 6 months after transplantation, most patients suffer from community-onset infectious disease because of maintenance minimal immunosuppressive therapy with good graft function.^[14]^ However, some studies have reported a trend toward later onset invasive aspergillosis in transplant patients.^[15,16]^ The time to first occurrence of IFI was longer in antifungal prophylaxis group, although significant difference was found. The changes of antifungal strategies in Korea seem to cause the delayed occurrence of fungal infection.

In this study, patients in non-prophylaxis group did not show unfavorable outcomes. This may be due to bias for patient selection, and further randomized controlled studies to analyze the effect of antifungal prophylaxis on clinical outcomes will be needed. However our result supports the use of targeted antifungal prophylaxis in high-risk patients

Raghuram et al reported that non-*albicans Candida* species accounted for 55% of IFIs, and only 43% of *Candida* isolates were fluconazole-susceptible in LT recipients.^[5]^*Candida* species were the predominant pathogen causing fungal infection in our study, with *C. albicans* constituting half of all *Candida* infections, and *C. glabrata* was found in only 1 case. Until now, our finding suggests that fluconazole-resistant *Candida* species have not emerged yet among LT patients in Korea, although we did not analyze the resistance pattern of *Candida* species. One study of invasive candidiasis in an Asia-Pacific area showed that non-*albicans Candida* species account for more infections than *C. albicans*, and fluconazole resistance was found in 18.2% of *C. tropicalis*, 5.2% of *C. glabrata* and in 2.2% of *C. tropicalis* isolates.^[17]^ There is an increasing trend of fluconazole resistance especially in non-*albicans Candida* species, and analyzing the resistance pattern in *Candida* species will guide antifungal drug choice for IFIs among LT patients in Korea.

Several studies reported that the risk factors for IFIs in LT patients were CMV disease, retransplantation, dialysis, fulminant hepatic failure, fungal colonization and prolonged operation time.^[2,7,18]^ A meta-analysis showed antifungal prophylaxis, especially fluconazole or liposomal amphotericin B, reduced the rate of IFI and IFI-related mortality.^[19]^ In our study, we also found that fungal colonization, retransplantation, and hyperalimentation were associated with IFIs among LT patients. Central venous catheterization was needed for hyperalimentation among LT patients, and catheter use may be a source of *Candida* infection. In addition, catheter-related fungal infection in LT patients occurred in 26% of cases, indicating the need for further efforts to decrease catheter-related infection.

Our study has some limitations. First, this is a retrospective study. There was lack of regional data for fungal complications among LT recipients in Korea. This study included large number of cases (482 LT patients) from 3 major transplantation centers in Korea, and our data could overcome the limitations associated with a single-center study. Second, the use of antifungal prophylaxis and treatment was physician dependent, and this could have influenced the risk factors or outcomes. However, there are some issues for antifungal prophylaxis in Korea because of insurance policies and cost, and it is notable that this study was reflects current trends in clinical practice. Third, this study did not evaluate the effect of antifungal treatment on outcome.

## 5 Conclusions

The incidence of late-onset IFIs is increasing in the antifungal prophylactic era, and fluconazole-resistant non-*albicans Candida* species have not yet emerged in Korea. IFIs remain a significant predictor of mortality in the prophylactic era although there has been decreasing trend towards incidence of IFI in Korea. Early identification of patients with high-risk for IFIs is crucial to decrease the mortality (especially in retransplantation), fungal colonization, and hyperalimentation cases.

## Author contributions

**Data curation:** Youn Jeong Kim, Sang Il Kim, Jong Young Choi, Seung Kew Yoon, Gun Hyung Na, Young Kyoung You, Dong Goo Kim, Myoung Soo Kim, Jae Geun Lee, Dong Jin Joo, Soon Il Kim, Yu Seun Kim, Sang-Oh Lee.

**Funding acquisition:** Sang Il Kim, Eungeol Sim.

**Investigation:** Youn Jeong Kim, Sang Il Kim, Jong Young Choi, Seung Kew Yoon.

**Methodology:** Myoung Soo Kim.

**Software:** Eungeol Sim.

**Writing – original draft:** Youn Jeong Kim.

**Writing – review & editing:** Sang Il Kim, Myoung Soo Kim, Jae Geun Lee, Dong Jin Joo, Soon Il Kim, Yu Seun Kim, Sang-Oh Lee, Shin Hwang.
